# Selections

**Published:** 1893-02

**Authors:** 


### *SELECTIONS AND ABSTRACTS.*

### Chancre of the Scalp.

At the 118th meeting of the N. Y. Dermatological Society (*Journ. Cut. and Gen. Ur. Dis.*, Jan., 1893) Dr. Vaughn presented a case of chancre of the scalp supposed to have resulted from a scratch by a syphilitic man during a fight. This situation is extremely rare, simply because it does not offer much exposure to infection. The occurrence of extra-genital chancre should not be lost sight of by the physician, as remembering it may aid him in diagnosing obscure sores and prepare him to combat the syphilitic symptoms following. M. B. H.

### Heroic Treatment of Chronic Gonorrhœa.

In the discussion of the treatment of chronic gonorrhœa in the male at the Atlanta Society of Medicine, January 17th, Dr. Willis F. Westmoreland reported a case in which there was no discharge so long as the patient remained quietly in bed, but getting up and out brought it on. Finally he told the patient to go to a dance one night, dance, drink all the champagne he could and finish the night with some woman of "easy virtue" and if that didn't cure him to get another doctor. The patient followed directions in the prescription to the extreme and had no further gonorrhœa. M. B. H.

### Syphilitic Nodes of the Hyoid Bone.

Dr. Geo. T. Elliot of New York reports (*Journ. Cutaneous and Genito-Urinary Diseases*, January, 1893) five cases of syphilitic nodes of the hyoid bone. There was severe pain upon speaking, swallowing or from some movements of the neck. Thorough examination of the hyoid region revealed one or more periosteal nodes on the bone, either alone or accompanied with a chondritis or epichondritis of the neighboring part of the thyroid cartilage. Laryngologists to whom he spoke had never observed this condition—hence its interest. He gives the clinical history of only two of his cases—these typifying the condition. There were or had been sufficient other manifestations of syphilis to confirm the diagnosis. The nodes disappeared under the use of iodide of potassium in the first case, and in the second mercurials, first inunctions, later calomel with iodide of potassium for exacerbation of nodes. A peculiar, effect of the iodide in the second case was, besides the ordinary mucous symptoms of iodism, a leucorrhœa, proven wholly due to the iodide. M. B. H.

---

### A CASE OF SKIN SHEDDING.

Dr. H. W. Blanc, of Sewanee, Tenn., reported at the Tri-State Medical Association, October, 1892, a case of skin shedding in the practice of Dr. Bolton of Biloxi, Miss. Patient, a young woman of twenty, had had three attacks—in 1890, in 1891, and in 1892—the one reported. Attack began with febrile symptoms but the temperature and pulse showed only slight elevation. At the end of about six days the erythematous eruption terminated in exfoliation of the mucous membrane of the roof of the mouth in a continuous sheet and from the body generally in large patches, while the epidermis of the hands and feet came away almost as perfect in shape as gloves and moccasins. The paper closes with the following parallel diagnostic table.

| SCARLATINA. | ERYTHEMA EXFOLIATION RECURRENS. |
|---|---|
| 1. Occurs once. | 1. Recurs frequently. |
| 2. Contagious. | 2. Non-contagious. |
| 3. Marked pyrexia. | 3. Pyrexia very slight. |
| 4. Fauces much swollen | 4. Fauces red. |
| 5. Pulse very rapid. | 5. Pulse not rapid. |
| 6. Eruption lasts five days. | 6. Eruption lasts three days. |
| 7. Desquamation sometimes considerable. | 7. Desquamation always excessive. |
| 8. Albuminuria common. | 8. No Albuminuria. |

M. B. H.

### THE TREATMENT OF PULMONARY TUBERCULOSIS APART FROM CLIMATE.

*The Times and Register*, Philadelphia, of January 6th, contains an article by Dr. Karl von Ruck, entitled as above, in which the author considers the various remedial agents apart from climate and urges their painstaking adoption both at climatic resorts and at places without the advantage of a favorable climate.

He describes the treatment so carried out in the special institution, the Winyah Sanitarium at Asheville, N. C., under his charge, and believes the same method can be carried out in private practice at least to a degree which would assure improvement and cure in a greater number of cases. In the treatment advised the patient is first instructed as to the danger from his expectoration and receives proper instruction as to its safe disposition. He is warned of the dangers of physical and mental overexertion and is told that, while exercise is essential and beneficial, it becomes indifferent or a source of danger and a cause for relapses when carried to a degree of sensible fatigue or when it is taken so rapidly that it produces shortness of breath. Fever is treated by absolute rest and proper diet, and if necessary stimulants and hydropathic applications. Drug anti-pyretics are entirely avoided. The diet for fever patients is light, in high fever solid food is entirely avoided; the feeding is frequent, and if the patients lose flesh under the regimen rectal feeding is resorted to in addition. Patients free from fever receive a generous mixed diet, well cooked, nicely served to the avoidance of pastries and articles of food which in the particular case seem to disagree.

In dilatation of the stomach and severe gastric catarrh, lavage and the use of electricity are recommended ; creasote is only given for its influence upon digestive derangements, and the author says it has no specific effect upon the tubercular process, having demonstrated that the germs grow luxuriantly in blood serum from patients who were practically saturated by large and long continued doses of creasote, and that the germs from their sputum will produce virulent cultures. As a general tonic and to prevent taking cold, every patient receives a cold water rub with brisk friction thereafter, and in a weak heart he uses strychnia in full doses.

Cough mixtures are never used; the general management being correct, there is no indication for them.

Local pleurisy is treated by rest and counter-irritation.

Urine analysis should certainly be made in all cases where the disease has advanced to destructive changes, amyloid kidney being more frequently found than text books indicate, and these cases are hopeless.

Bloody expectoration is treated by rest, and cough then moderated by codeine. Expectoration of clear blood and more decided hemorrhage occurred in less than one per cent. in his private institution and also much less frequently in his private practice since he has learned to appreciate the detrimental effects of over-exertion. He distinguishes the form due to destructive changes and that which results from overexertion, rest, diet, ice, morphia, strychnia and stringent inhalations being considered the most useful.

The author still believes in tuberculin, although he was obliged to abandon its more general use in his institution owing to prejudice both of the profession and of patients.

He never saw any disagreeable effects; on the contrary he witnessed most satisfactory improvement under its use as advised by him.

The pneumatic cabinet is used in a restricted sense and good results as to better circulation and vital capacity have been observed.

Oxygen and ozone are used frequently in connection with ferruginous preparations when anæmia does not yield to the climatic and other influences.

Plenty of outdoor life is recommended both at home and at the resort.

The paper being based upon a large experience, can be recommended for study to any physician who treats consumptives.

### LAWSON TAIT ON THE TREATMENT OF PERITONITIS.

Years ago I was driven to the determination to discontinue the use of opium by the mouth after abdominal operations, for two reasons: first, its action on the intestines is most certainly to modify and even suspend vermicular movements; and secondly, it masks the real condition of the patient. One dose of morphine, given under the skin immediately after the operation, is all that my patients ever get, and the bulk of them do not get that.

Regarding the intolerable thirst that follows the opening of the peritoneal cavity, I have come to look upon it not as an indication for the administration of fluids, but rather for withholding them. Ice is one of the things that should be banished absolutely from the sick-room. It never acts in any other way than to increase thirst.

Again, if nausea sets in on the third or fourth day, or at any time after, all food (and in that I include water) is absolutely stopped for twelve hours or even longer if necessary. I was driven to this by the uniform experience that all drugs employed for the arrest of vomiting were absolutely futile; that any food given came back only altered by biliary admixture. Further, I was influenced by the perfect certainty that nothing could possibly be digested or absorbed by the stomach so long as bile was being poured into it.

I have therefore a belief that the starvation and withholding of fluid prevents the mechanical stasis of the circulation in the intestinal coats, which appears to me to be the initial stage of the fatal process of peritonitis, and this preventive measure I endeavor to assist by stimulating the peristaltic movement. I have tried a vast number of different kinds of enemata--some suggested by own thought and others suggested by ingenious friends—but I have always gone back to soap and turpentine. It is the business of any nurse watching one of my abdominal sections to note every six hours a set of four conditions—the pulse, the temperature, the occurrence of distension, and the passage of flatus *per anum*. So soon as the latter is freely and naturally established our anxiety ends, though our watchfulness is unremitting. The want of such passage for twenty-four hours after an operation, especially if accompanied by the slightest suspicion of distension, is dealt with without fail by the nurse herself, on her own responsibility, by the administration of a turpentine enema. If the turpentine does not answer the nurse reports, and a mild saline purgative is ordered—generally a seidlitz powder—and this is repeated every four hours until it acts. If the distension increases we never rest until we have had the bowels moved, and then our anxiety is nearly always at an end and our efforts rewarded by recovery. But above all things there must be no time lost, and nobody who may be called into consultation by anxious friends must be permitted to write a prescription and try some favorite mixture.

When called in to a case of well-established peritonitis I always urge a trial of this treatment, because the stage may not have passed at which it may still be effective, but the chances are that it has. I have never said that the purgative treatment will cure peritonitis, for peritonitis, once it is completely established, is a practically incurable disease, and almost uniformly fatal. Of this I am certain, if you subject the patients (not the peritonitis) to the purgative treatment, the number who will go on to incurable peritonitis will be fractional compared to what will be the result if they are left alone or submitted to any other treatment.

The practical outcome of my empirical experience is this, that the purgative treatment of peritonitis or threatening peritonitis, if it be promptly brought to bear on the case, will gain the all-important time, which will eventually turn the scale in the favor of the patient.—*British Medical Journal.*

---

### CARDIAC TONICS.

At a late meeting of the British Medical Association, an interesting debate was held respecting the action of the cardiac tonics, digitalis, strophanthus, convallaria, caffein and spartein ; digitalis being taken as representative of the class. Dr. Broadbent said (in substance) the physiological action of this drug is a direct stimulation of the muscular fibres of all the cardiac-vascular system, improving the contractility of the heart, arterioles and capillaries, independently of any intermediate nervous influence. The therapeutic value of this property is evident when the transit of blood through the heart is hampered by disease. The ventricles, by their improved contraction, help to fill the arterial side of the circulatory system; and improved suction power during diastole helps to empty the veins and relieve the venous plexuses and abdominal organs. The systole is also improved, and the heart invigorated by the physiological rest it obtains through prolongation of the diastole. The improved contraction of the arterioles and capillaries hastens the blood-current, assisting the propulsive force of the heart, better than could be done by flaccid dilated vessels, and favoring the absorption of effused fluids. In mitral regurgitation digitalis is always of great service. The returning blood dilates the left auricle, and drives the flowing current back again. This obstruction causes resistance to the flow through the capillaries, and to overcome this, the right ventricle has to work with increased vigor, causing hypertrophy. Again, distension of the left ventricle, by the high pressure of the left auricle and pulmonary veins, causes dilatation, thus in mitral regurgitation the compensation has to be supplied by the right ventricle; and it is this portion of the heart which is amenable to the action of digitalis, for this drug always neutralizes the effects of regurgitation, unless the right ventricle is seriously disabled by disease. In mitral stenosis digitalis may do harm, for the vessels are already too much contracted and the remedy would increase the evil. Sometimes it does a little good at first by contracting the right ventricle, but it disorders the heart beats, and its power for evil counterbalances the good. In aortic regurgitation the blood is either defectively propelled into the arteries, or there is obstructive back-working through the lungs and right heart, causing venous obstruction and dropsy. When the blood is defectively propelled digitalis is not beneficial; but in the other case it is of decided value, especially when there is dropsy. In aortic stenosis, when there is back pressure in the pulmonic and venous circulation, through dilatation of the left ventricle, digitalis is serviceable, but when the disease is due to a limited supply of arterial blood, through obstruction, it does harm. In dilatation of the heart digitalis does good when the walls of the ventricles are in fair condition, not otherwise. Blue mass and calomel are here more appropriate, by unloading the liver, relieving the right heart and lowering arterial tension. In adherent pericardium—a not infrequent result of mitral disease—the right ventricle is impeded from lending assistance to the left, symptoms may become prematurely developed, often causing sudden death. Cardiac tonics are here of little use. Digitalis is very beneficial in the weak heart of typhoid fever and pneumonia, but in Graves' disease, tachycardia, and in some forms of palpitation and irregular pulse due to reflex disturbances, it is useless. Strophanthus is preferable to digitalis only when the arterioles need no stimulation. Citrate of caffein increases the solids of the urine. It is often a useful adjuvant to digitalis. The same may be said of spartein. Convallaria is valuable in nervous palpitation.—*Times and Register.*

---

### ANTIPYRINE, PHENACETINE, PHENOCOLL.

Drs. David Cerna and Wm. S. Carter, of the University of Pennsylvania, have reported a large number of interesting experiments to determine the comparative actions of these three agents on the circulation and heat phenomena. They arrive at the following conclusions :

1. Antipyrine, phenacetine and phenocoll all fail to produce any effect on the heat functions of the normal animal.

2. Antipyrine produces a decided fall of temperature in the first hour after its administration in the fevered animal. This reduction is due to a great increase in heat dissipation, together with a fall in the heat production.

3. Phenacetine, both in septic and albumose fevers, produces a very slight fall of temperature during the first and second hours after its ingestion by the stomach, but the greatest reduction occurs the third hour after its ingestion. The fall of temperature results chiefly from a decrease in heat production, with a slight increase in the heat dissipation. The increase in dissipation is not as great as with antipyrine. Probably the delayed action of the drug depends on its insolubility.

4. Phenocoll causes in fever a very decided fall in temperature, which occurs the first hour after the administration of the drug by the stomach. This reduction is the result of an enormous diminution of heat production, without any alteration of heat dissipation.

Our experiments with antipyrine, in accord with the results obtained by Martin, Wood, Reichert and Hare, together with Destree, have reached the conclusion that antipyrine reduces the temperature by a decrease in heat production, and that heat dissipation also falls with the production.

In our experiments with antipyrine the composite curve shows the rise of heat dissipation. We believe, therefore, that this phenomenon is effected through a thermotaxic rather than through a thermogenic mechanism. We further believe that phenacetine and phenocoll reduce the temperature by a decrease in the heat production through their action on a thermogenic nervous center. The fact that all drugs here studied fail to produce any effect on the normal heat function proves that they affect these functions through the nervous system. Probably the fact pointed out by Hare, in his excellent essay, that many investigators do not take into account other circumstances, such as tying animals down and confinement in a box, may explain many of the results obtained by some observers in the normal animal.

In concluding this study we are justified, judging from the results of our experimentation, in saying that of the three drugs in question, the safest for practical purposes, especially as regards an action upon abnormal temperatures, would be phenocoll. Phenacetine is slow on account, no doubt, of its insolubility, and is comparatively feeble as an antipyretic. Antipyrine, it is true, is soluble and prompt in reducing feverish conditions, but its action upon the circulation, particularly upon the heart, is so pronounced, even when administered in therapeutic doses, that it is for this reason a dangerous substance to use. Phenocoll, on the other hand, is readily soluble, rapidly absorbed and undoubtedly promptly eliminated. Its power to reduce abnormal high temperatures is very decided, and it does this in therapeutic doses without depressing the circulation. Phenocoll, therefore, would seem to be superior to antipyrine and phenacetine as an antipyretic.—*Ohio Medical Journal.*

---

### THE TREATMENT OF TYPHOID FEVER.

The treatment of typhoid fever at the Philadelphia University Hospital is as follows:

1. Milk diet.

2. Absolute rest in bed; enforced use of bed-pan and urinal. Disinfection of stools with chloride of lime.

3. Tub bath whenever temperature reaches $102\frac{1}{5}°$.

### RULES FOR BATHING.

(*a*) When temperature of patient is below 101°, temperature observation to be made every three hours; when above 101°, every hour; when 102°, every half hour.

(*b*) Temperature of bath, 70°.

Tub is wheeled aside the bed and patient lifted in by two nurses, one holding the patient under the arms, the other by the legs.

(*c*) Systematic rubbing is kept up during the bath, and afterwards if the patient shivers much.

(*d*) Water (temp. 50°) is poured over head and shoulders at the beginning of the bath.

(*e*) If desirable, a hot drink, such as hot milk or milk punch, may be given the patient during the bath. If temperature is difficult to reduce, a glass of ice-water may be given instead.

(*f*) Patient remains in bath from fifteen to eighteen minutes. If temperature tends to remain high, cold cloths or an ice-cap are applied to the head.

Above is the detail of the bath treatment used here. No medicines are given constantly. The vast majority of cases require whiskey in greater or less quantities.

Indications are treated as they arise. Our rule is to keep patient in bed and upon milk diet for one week after the temperature reaches normal.

In regard to the prevalence of typhoid fever since September 1st, and the locality of the residences of the patients, I would state that we have had ten cases of it in the University Hospital since the above-mentioned date, and that six of these have resided in West Philadelphia within a radius of one mile of the hospital.

The cases have been for the most part uncomplicated, and our death rate has been *nil.*

In one case a relapse occurred on the thirty-first day, after an apyretic period of seven days. This occurred despite the fact that the patient occupied still the dorsal decubitus, and was still on a milk diet.

The treatment pursued at this hospital is the Brand treatment. Whenever the temperature of the patient reaches $102\frac{1}{5}°$ he is plunged into a tub of water (temperature of 70°) and allowed to remain for fifteen minutes. The effect of the baths on the temperature of different patients varies. Some drop immediately to normal or thereabouts, while others require a repetition of the baths to keep the temperature below $102\frac{1}{5}°$. In none of the bath cases was there observed any delirium whatsoever.—*J. F. Schamberg, M. D., Resident Physician, University Hospital, in Times and Register.*

---

### STATISTICS OF PULMONARY TUBERCULOSIS.

The statistics are taken from 500 consecutive cases of pulmonary tuberculosis treated by the writer at St. Andreasberg, in the Hartz mountains, in the years 1884–90. In 26.3 per cent. of 475 cases one or both parents were tuberculous ; 11.8 per cent. of the children of patients were tuberculous. In 13 instances infection from husband to wife or *vice versa* was highly probable. Fifty-nine per cent. of the cases were male, 41 per cent. female. Fifty per cent. of the cases occurred between the ages of 20 and 30. Between the ages of 15 and 20 the percentage of cases was nearly twice as great among males as females. The duration of the disease is proportional to the patient's age and is longer in males than in females, averaging 4 years 5 months in the former, and 3 years 9 months in the latter. Chill was given as the cause in 33 cases. In 28 cases the disease followed a pleurisy, and in them the mortality was 43 per cent., while in 19 cases in which the disease developed after pneumonia, the mortality was but 2.2 per cent. Hæmoptysis was the first symptom noted in 33 cases (32 of whom were males) ; in 13 of them it occurred without apparent cause ; probably cases of previously latent tuberculosis ; in some of the remaining cases the hæmoptysis may be regarded as the cause of the disease, as in 12 cases where it followed excessive bodily exercise by apparently healthy persons. Hæmoptysis was noted in the course of the disease in 35.6 per cent. of the cases. The mortality in these cases had been but 0.8 per cent. greater than in the other cases. Rectal fistulæ were noted in 9 cases, in 2 of which they had existed a long time before other symptoms. The etiological influence of frequent childbirth, severe puerperal disease and prolonged nursing is illustrated. In 43.8 per cent. of cases of catarrh of the apex, tubercle bacilli were found in the sputum. The lower lobe was primarily affected in 11 cases. At the end of treatment, the duration of which varies considerably, 92.6 per cent. were undoubtedly improved ; 2.6 per cent. remained about the same, 1.8 per cent. grew worse ; and 3 per cent. died while under treatment. The value of elevation in the treatment of phthisis is illustrated by the high percentage of improvement. Information has been obtained from 419 cases : 168 have died (3 of intercurrent disease) ; 32 temporarily improved are approaching a fatal termination ; 79 remain so much improved that they attend to their business ; and 125 may be considered as cured, as they enjoy the best of health and have suffered no serious relapse.—*Prager Med. Wochenschr.*—*Epitome of Medicine.*

### THE CURABILITY OF ASCITES.

It is necessary chemically to divide all cases into two classes, viz.: those in which the liver is enlarged and those in which it is not. The prognosis in the former category is by no means hopeless. Cheadle collected thirty-three cases of recovery, and in all of them in which the size of the liver is noted the organ was enlarged. The two great factors in the production of hepatic cirrhosis are alcohol and syphilis, the former being the most frequent agent in determining the inflammatory lesions which culminate in cirrhosis, but, as is pointed out, there is a certain social relationship between alcholism and syphilis which renders it difficult to draw a sharp line so far as the etiology is concerned. Turning to treatment, we find it has not as yet been markedly influenced by the advances in our knowledge of the morbid anatomy of this affection. The dreary routine of purgatives and diuretics with paracentesis as a last resort, has been maintained to the prejudice of the patients and to the despair of the physician. The patient is already extremely debilitated by the disorganization of his internal economy, and to administer to such a person drastic purgatives with tedious reiteration is to do him more harm than good. As to diuretics they are inoperative, because the pressure of the contained fluid on the kidneys has disorganized the urinary service and incapacitated these organs from responding to stimuli. Remove the pressure by means of paracentesis and then diuretics will act, but not otherwise. There is, to be sure, the exclusive milk diet, for which admirable results are claimed by Professor Semmola in the curable class of cases, but as it requires to be enforced not for weeks, but for months, the patients are apt to opine that the remedy is worse than the disease, and to kick over the traces. Dr. Cheadle is strongly in favor of tapping early and tapping often. By relieving the intra-abdominal pressure, the best chance is afforded for relief to the congested portal circulation by the establishment of a collateral circulation through the venous anastomoses. Collapse need not be feared if paracentesis be performed early; peritonitis may be avoided with certainty if the trocar be aseptic, and the patient is spared the visceral complications which are the inevitable consequence of the continued presence in the abdomen of a large quanity of fluid at high pressure.—*Eng. Med. Press.*

---

### GUNSHOT WOUNDS IN THE ABDOMEN.

There are four cardinal points in the management of the abdomen, viz.:

1. Have everything in readiness, the patient thoroughly prepared, the abdomen thoroughly cleansed, and the surrounding surfaces covered with antiseptic cloths before an anesthetic is administered. I have seen patients kept under an anesthetic for ten or fifteen minutes while the operator and assistant were getting things ready. This materially lessens the chances for recovery, for it is well known that the shorter the period of anesthesia, the less the shock, etc.

2. After the abdomen shall have been opened the first business in hand should be to find the source of the hemorrhage, if any, and check the same. I speak from sad experience on this point, for I believe I lost a patient from lack of observance of this rule. While sewing up gunshot holes in the small intestines, which were not bleeding, and the closing of which could just as well have been delayed, a fatal hemorrhage was going on at another point.

3. As far as possible the intestines should be kept in the peritoneal cavity. I know, however, that this cannot always be done. All know that useless handling of the gut, and the dragging upon its mesentery, as well as the exposure to cold, etc., adds greatly to the shock.

4. To finish the operation at as early a moment as possible, consistent with the proper management of the same.

It is impossible to lay down hard and fast rules for the government of the surgeon in his dealings with these cases, as the difficulties in each must be surmounted as they arise.—*Dr. H. C. Dalton in Annals of Surgery.*

